# Sex Differences in Vascular Compliance in Normal-Weight but Not Obese Boys and Girls: The Effect of Body Composition

**DOI:** 10.1155/2012/607895

**Published:** 2012-03-11

**Authors:** Jeanie B. Tryggestad, David M. Thompson, Kenneth C. Copeland, Kevin R. Short

**Affiliations:** ^1^Section of Diabetes and Endocrinology, Department of Pediatrics, University of Oklahoma Health Sciences Center, Oklahoma City, OK 73104, USA; ^2^Department of Biostatistics and Epidemiology, University of Oklahoma Health Sciences Center, Oklahoma City, OK 73104, USA

## Abstract

*Objectives*. To determine the effect of sex and obesity on vascular function in children and explore potential mechanisms that account for differences in vascular function. *Methods*. Participants were 61 (30 boys) normal-weight (BMI 25–75% ile for age and sex) and 62 (30 boys) obese (BMI ≥ 95% ile) children of ages 8–18 years. Measurements of large and small artery elastic index (LAEI and SAEI, resp.) and reactive hyperemia index (RHI) were obtained at rest, along with anthropometric and biochemical information. *Results*. In normal-weight children, LAEI was 16% higher in males than females (*P* = 0.04) with a similar trend for SAEI (13% higher in males, *P* = 0.067). In obese children, no sex-related differences in vascular measures were observed. In multivariable models, sex differences in arterial compliance were explained by higher lean mass in normal-weight boys. Fat mass predicted LAEI and SAEI in both normal-weight and obese females, but fat mass predicted arterial compliance in boys when fat mass exceeded 24 kg (37% of the sample). *Conclusions*. Normal-weight males have higher arterial compliance than normal-weight females due to increased lean mass, but sex-related differences were not observed among obese children due to a lack of sex-related differences in lean or fat mass.

## 1. Introduction

 Cardiovascular disease has been the leading cause of death in the United States for the past 80 years [1] and results in 40% of all-cause mortality in developed countries [2]. Men have a higher prevalence of cardiovascular disease than women due in part to earlier disease onset, although women have a disproportionate increase in cardiovascular disease incidence after menopause due in part to the cardioprotective influence of estrogen [2, 3].

 Atherosclerosis begins in childhood [4] and slowly progresses toward symptoms of cardiovascular disease such as angina and myocardial infarction [5]. Impaired vascular endothelial function, measured by forearm reactive hyperemia or flow-mediated dilation, is an early sign of vascular risk that is associated with abnormal coronary angiographic findings in children [4]. Low arterial compliance, another index of vascular function, has also been associated with microvascular disease and contributes to end-organ damage, stroke, and renal damage in adulthood [6]. Obese adults have lower arterial compliance [7] as well as impaired endothelial function compared to normal-weight controls [8]. Paradoxically, recent studies [4, 9, 10] have shown that arterial compliance is higher in obese children, likely attributable to greater lean and fat mass in obese children compared to their normal-weight peers [10].

 Differences between boys and girls in arterial compliance and endothelial function have been reported, but it is not yet known whether these differences can be ascribed to variation in body size and composition or if there is a specific effect of obesity. Two studies in children reported that PWV was higher in boys than girls and positively related to height and blood pressure [11]. There may, however, also be sex differences due to developmental stages and hormonal changes. Ahimastos and colleagues found that boys had lower PWV (higher arterial compliance) than girls before puberty, but these differences were no longer evident in postpubertal adolescents [3]. A causal link was not established [3], but the authors proposed a sex hormone influence on vasculature, mediated potentially via endothelial nitric oxide synthase (eNOS). Thus, the existing data support the presence of sex differences in vascular function that begin to appear during childhood. None of the existing studies, to our knowledge, has considered whether sex and obesity interact in their effects on vascular function children. This question is of particular importance because of the rapid increase in childhood obesity and its potential consequences for cardiovascular disease risk.

 We previously demonstrated that arterial compliance is positively associated with lean body mass in normal-weight and obese children and associated with fat mass in obese children [10]. The purpose of the present investigation was to test the hypothesis that differences in vascular function during childhood vary with sex and obesity status. This goal was accomplished through analyses of arterial compliance and vascular reactive hyperemic responses in obese and normal-weight boys and girls. We also assessed whether sex differences in these outcomes were attributable to variations in age, maturation (Tanner stage and height), body composition, or biochemical factors.

## 2. Methods

### 2.1. Subjects

 One hundred twenty-three healthy boys and girls of ages 8 to 18 years old participated, including 30 obese boys, 32 obese girls, 30 normal-weight boys, and 31 normal-weight girls. Obesity was defined as having a body mass index (BMI) greater than the 95th percentile based on Center for Disease Control (CDC) growth charts. The criterion for inclusion in the normal-weight group was a BMI within the 25–75th percentile. Children with known diabetes, cardiovascular disease, or other chronic debilitating diseases were excluded from the study.

 Informed written consent and assent were obtained in accordance with the guidelines of the University of Oklahoma Health Sciences Center Institutional Review Board for Human Subjects.

### 2.2. Anthropometric Measures

 A routine medical history and physical examination were performed by a pediatrician, and Tanner staging was determined for pubertal status. Body composition was quantified using dual energy X-ray absorptiometry (DXA, GE iDXA, Fairfield, CT).

### 2.3. Arterial Elasticity

 Elasticity of the large and small arteries was measured using diastolic pulse-wave analysis (HDI/Pulsewave CR-2000, Hypertension Diagnostics, Inc, Eagan, MN), as described previously [10]. After resting supine for 10 minutes, an upper arm blood pressure cuff was placed on one arm, a wrist stabilizer was placed on the opposite wrist, and a tonometer was placed over the radial artery to obtain pressure waveforms. The HDI device recorded arterial waveforms for 30 seconds, digitized and stored 200 samples per second, and then calculated small and large arterial elasticity (SAEI and LAEI, resp.) from the sampled pulse wave contour. The mean of three replicates for each participant was used for data analyses.

### 2.4. Arterial Reactive Hyperemia

 Fingertip reactive hyperemia was measured using peripheral arterial tonometry (Endo-PAT2000, Itamar, Franklin, MA). Plethysmographic sensors were placed on the index finger of each hand, and pulse pressure amplitude was recorded during a 5 minute baseline, during 5-minutes of cuff occlusion of the upper arm, and during 5 minutes of postocclusion recovery. The reactive hyperemia index (RHI) was calculated as the ratio of the average pulse amplitude measured over 60 seconds, starting 1 minute after cuff deflation, to the average pulse amplitude measured at baseline. The ratio was corrected for changes in systemic vascular tone by normalizing the results to the contralateral, nonoccluded arm.

### 2.5. Serum Analysis

 Glucose was measured by glucose oxidase method (YSI 2300 STAT plus, Yellow Springs, OH). Insulin was measured using Human Insulin ELISA kit from Millipore (St. Charles, MO). Glucose and insulin values were used to calculate fasting insulin resistance using the HOMA-IR model [12]. C-reactive protein (CRP) and lipids (total cholesterol, LDL-cholesterol, and HDL-cholesterol, and triglycerides) were measured by the Veteran's Administration Hospital Clinical Laboratory in Oklahoma City, OK, USA.

### 2.6. Statistical Analysis

 Descriptive statistics including mean, standard deviation, and standard error of the mean were calculated on all outcomes. After data were inspected for normality, *t*-tests were used to quantify differences in anthropometric, serum, and arterial function measurements between boys and girls groups. Univariate and multivariate modeling using linear regression and analysis of variance (SAS version 9.1, Cary, NC) were used to establish associations between the arterial function measures and the anthropometric and serum data. 95% confidence intervals were calculated on all significant models. Nonlinear piecewise regression models were used to explore in detail the association between arterial compliance and fat mass over the range of observed values for fat mass.

## 3. Results

### 3.1. Anthropometric Data (Table 1)

 In both normal-weight and obese groups, the numbers of boys and girls were similar. Within the normal-weight group, the boys had greater lean mass (*P* < 0.01) and less fat mass than the girls (*P* < 0.01) although Tanner stage and blood pressure were not different. In the obese group, the girls were more advanced in pubertal staging (*P* < 0.01), but the sexes did not differ for body size, body composition (body fat or lean mass), or blood pressure.

### 3.2. Serum Biochemical Data (Table 2)

 No differences between boys and girls in the normal-weight group were observed in any of the measured serum variables. However, the obese boys had higher triglycerides and lower insulin compared to the obese girls (*P* < 0.05). There was no difference in total cholesterol, LDL-C and CRP confirmed by non-parametric tests and *t*-tests performed on log transformed data.

### 3.3. LAEI (Figure 1)

 In the normal-weight group, mean LAEI was 16% (2.20 mL/mmHg × 10) higher in boys than girls (*P* = 0.04; 95% CI: 0.10, 4.32), although in the obese group, boys did not differ from girls in LAEI. To account for the potential differences in adiposity between boys and girls, regression models for LAEI were adjusted for fat mass in the normal-weight group, and mean adjusted LAEI remained significantly higher in boys by 3.85 mL/mmHg × 10 (95% CI: 1.79, 5.92; *P* ≤ 0.01).

 Within each of the four groups, several measures correlated positively with LAEI.  Table 3 details the significant univariable correlations with LAEI which were used for establishing multivariable models.

In multivariable models with regard to normal-weight boys, either height (*r* ^2^ = 0.52) or lean mass (*r* ^2^ = 0.47) alone best predicted LAEI; adding other variables resulted in overfitting the model. For every 1 cm increase in height, LAEI increased 0.17 mL/mmHg × 10 (95% CI: 0.11, 0.23; *P* ≤ 0.01) in normal-weight boys. For every 1 kg increase in lean mass, LAEI increased 0.24 mL/mmHg × 10 (95% CI: 0.14, 0.34; *P* ≤ 0.01).

 In normal-weight girls, only single variable models were statistically significant in predicting LAEI. For every 1 cm increase in height in the normal-weight girls, LAEI increased 0.16 mL/mmHg × 10 (95% CI: 0.09, 0.24; *P* ≤ 0.01). For every 1 kg increase in lean mass, LAEI increased 0.31 mL/mmHg × 10 (95% CI: 0.15, 0.47; *P* ≤ 0.01), and for every 1 kg increase in fat mass, LAEI increased 0.48 mL/mmHg × 10 (95% CI: 0.23, 0.75; *P* ≤ 0.01).

 The multivariable model for the obese boys that best predicted LAEI included both age and fat mass (*r* ^2^ = 0.53). When age was held constant, LAEI increased 0.19 mL/mmHg × 10 for every 1 kg increase in fat mass (95% CI: 0.05, 0.33; *P* = 0.01). When fat mass was held constant, LAEI increased 1.60 mL/mmHg × 10 for each year of age (95% CI: 0.78, 2.42; *P* ≤ 0.01).

 In the obese girls, no multivariable model was superior to fat mass in predicting LAEI. For every 1 kg increase in fat mass, LAEI increased 0.24 mL/mmHg × 10 (95% CI: 0.09, 0.39; *P* ≤ 0.01).

### 3.4. SAEI (Figure 1)

 Normal-weight boys had 13% higher SAEI versus normal-weight girls (*P* = 0.067). Among boys and girls of normal-weight, SAEI was not significantly associated with any anthropometric or serum measure.

 In contrast, in the obese boys and girls, several measures were associated with SAEI.  Table 4 details the univariable correlations with SAEI that were used to construct the multivariable models.

In multivariate models for obese boys, the best prediction sets for SAEI contained fat mass and height (*r* ^2^ = 0.54) or fat mass and lean mass (*r* ^2^ = 0.54). In those models, fat mass, lean mass, and height were all independent predictors of SAEI. If height was held constant, SAEI increased 0.12 mL/mmHg × 100 for every 1 kg increase in fat mass (95% CI: 0.05, 0.19; *P* ≤ 0.01). Holding lean mass constant, SAEI increased 0.11 mL/mmHg × 100 for every 1 kg increase in fat mass (95% CI: 0.04, 0.18; *P* ≤ 0.01). The best model for predicting SAEI in obese girls was one that included only Tanner stage (*r* ^2^ = 0.31).

### 3.5. Role of Fat Mass in Arterial Compliance

**Figure 2:**
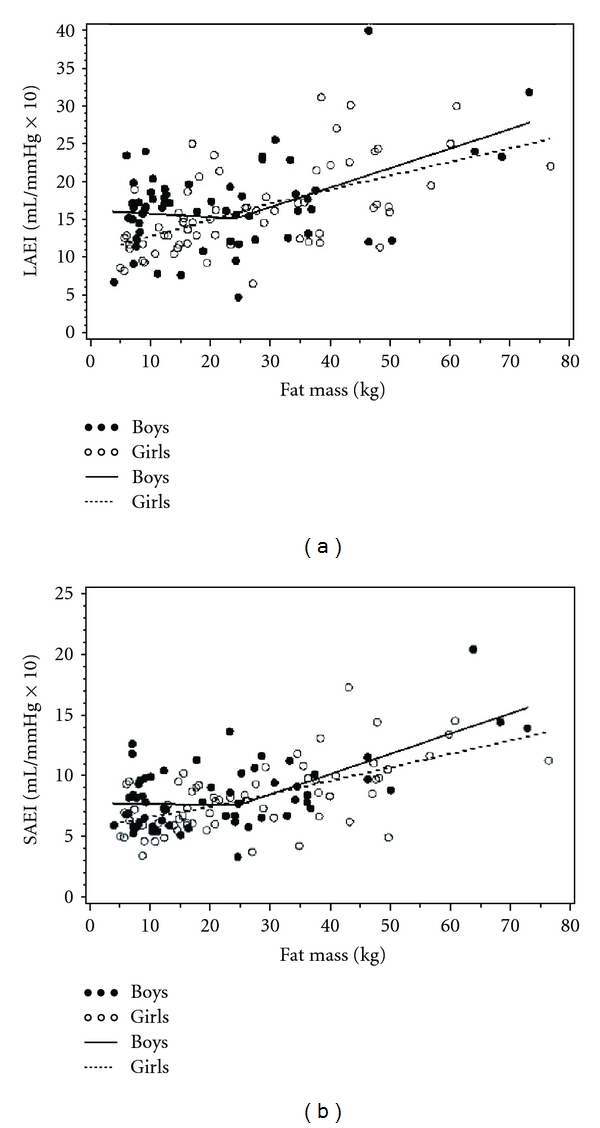
Association between total body fat mass and large arterial elasticity index (LAEI (a) and SAEI (b), resp.) in boys and girls. SAEI and LAEI were positively associated with fat mass in girls in a continuous fashion, while in boys, it was only positively associated with SAEI and LAEI after approximately 24 kg of fat mass.

Because fat mass predicted arterial compliance in the obese boys and girls, we investigated the role of fat mass in more detail in both normal-weight and obese children. Using piecewise nonlinear regression, we found that the linear relationship between fat mass and both LAEI (Figure 2(a)) and SAEI (Figure 2(b)) was discontinuous in boys, but continuous in girls. Among boys, fat mass was positively correlated with LAEI for values of fat mass greater than 24.3 kg (95% CI: 7.7, 41.0; *P* ≤ 0.01). Similarly, fat mass was positively correlated with SAEI for values of fat mass greater than 24.7 kg (95% CI: 13.1, 36.4; *P* ≤ 0.01). Thirty seven percent of the boys in the study had fat mass values greater than these cut points.

### 3.6. RHI (Figure 1)

 RHI did not differ between boys and girls within either the normal-weight or obese groups. No significant associations were found between RHI and the anthropometric and serum measures in boys. In girls, RHI in the normal-weight group was correlated with age (*r* = 0.60, *P* = 0.0004), Tanner stage (*r* = 0.56, *P* = 0.003), fat mass (*r* = 0.48, *P* = 0.007), and height (*r* = 0.43, *P* = 0.016), but in obese girls, no significant associations were found with the anthropometric outcomes. There were no significant correlations between RHI and serum outcomes in girls.

## 4. Discussion

 The goal of the study was to determine whether vascular compliance and endothelial function differ in boys and girls, and if these differences are affected by obesity. The main findings were that normal-weight boys had significantly higher large artery compliance and nonsignificantly higher small arterial compliance than girls, and that the differences were best explained by the boy's greater lean body mass. This difference in LAEI between normal-weight boys and girls was magnified when fat mass was statistically controlled. In contrast, in the obese group, boys and girls did not differ in either large or small arterial compliance. Unlike their normal-weight peers, the obese boys and girls did not differ in lean and fat mass; this lack of difference in body size and composition appears to explain their similar vascular compliance.

 Another novel finding was that fat mass correlated differently with vascular compliance in boys versus girls. In girls, fat mass correlated positively with vascular compliance in a linear fashion; however, in boys, there appears to be a threshold effect of fat mass (~24 kg) below which there is no relationship and above which fat mass is positively associated with LAEI and SAEI.

 There was no difference in RHI between boys and girls in either the normal-weight or obese groups. We found that age, height, and Tanner stage were predictive of RHI in the normal-weight girls, but no variables predicted RHI in boys or in obese girls. The association of RHI with age, height, and Tanner Stage in girls may be related to changes in estrogen during puberty. In young adults, brachial artery dilation was greater in women even though women had smaller vessels than men [13]. The authors suggested that estrogen-stimulated NO production may explain the differences between sexes and may explain the increase in RHI with maturation in females [2].

 The present study demonstrates that both lean and fat mass are significant predictors of both small and large artery compliance and that this relationship may be stronger in obese children than in normal-weight children. We recently reported that body composition, particularly lean mass determined by DXA, was as good or better than height for predicting LAEI and SAEI in children [10]. The lack of difference in lean and fat mass between the obese boys and girls could explain why those groups had similar arterial compliance.

 A novel finding of this report is that the pattern of association between fat mass and vascular compliance differed in boys and girls. Fat mass was positively associated with compliance in girls across the range of fat mass (5.2–76.4 kg). In boys, the association between fat mass and compliance was not evident until fat mass surpassed approximately 24 kg, when it was positively correlated (overall range of fat mass in boys was 4.2–72.9 kg). While the mechanism underlying the boy's discontinuous response is unclear, we speculate that the vascularity of adipose tissue, or its production of vasoactive compounds, must reach a higher threshold in boys than girls before a measureable impact on vascular function is observed.

 One of the factors that may contribute to the association of fat mass with arterial compliance is that adipocyte-related hormones or cytokines from the fat tissue may promote increased arterial compliance (elasticity) in obese children. A potential mechanism that could support this premise is an increase in vascular endothelial nitric oxide synthase (eNOS), leading to increased nitric oxide, greater smooth muscle relaxation, and increased arterial compliance [14]. At least two adipocytokines, leptin and visfatin, as well as insulin, are elevated in obesity and are known to increase eNOS [15–21]. In adults, both eNOS protein and mRNA content are higher in the subcutaneous fat of obese individuals compared to normal-weight controls [22, 23] although, critically, NO bioavailability is decreased in obese adults [24]. How this occurs is not yet known. In early type 1 and type 2 diabetes, basal NO is increased early in pathogenesis, but then decreases as microvascular disease progresses [25]. It is therefore possible that insulin and leptin promote eNOS synthesis and NO production early in obesity, but over time, the effect of the reactive oxygen species prevails. Further research can explore the mechanisms that explain the paradoxical differences in the effect of obesity on vascular compliance in children and adults.

 Estrogen is another vasoactive hormone that may explain the differences between boys and girls. Estrogen increases eNOS and NO production [2] providing a plausible link between fat mass and arterial compliance in the present study, particularly in girls. Our finding of a positive correlation between Tanner stage and RHI in girls also points to a role for the increase in estrogen during development.

 The discontinuous relationship that we observed between fat mass and arterial compliance in boys may also be linked to the role of estrogen. Adipose tissue contains both aromatase and 17*β*-steroid dehydrogenase, which aromatize androstenedione to estrogen [26]. Plasma estrogen concentration is elevated in obese men as a result of upregulation of these two-estrogen producing enzymes [26–28]. In boys, we speculate that the threshold of ~24 kg of fat mass may be the point at which aromatase activity within the fat is sufficient to produce the observed positive association between fat mass and arterial compliance observed.

 Some limitations of the present study are acknowledged. This paper addresses secondary hypotheses that were suggested in the course of an earlier study [10]. The study's additional comparisons may elevate the overall probability of a type I error, thus declaring a difference to be significant when in truth it is not. We recognize this limitation and so have focused less on hypothesis tests and *P* values than on the size of the plausible effects and differences this study reports, as quantified in confidence intervals. Another potential limitation is the lack of hormonal data to support the proposed mechanisms for the differences or lack in boys and girls vascular compliance. The current data provides preliminary support for additional mechanistic investigations to explain the sex differences observed.

## 5. Conclusion

 In conclusion, the results of the current study demonstrate a difference in large artery compliance in normal-weight boys and girls that is not evident in obese children. The sex differences in arterial compliance in normal-weight children can be accounted for by differences in lean body mass. Although arterial compliance did not differ in obese boys and girls, it was still associated with lean and fat mass as it was in normal-weight children. We also demonstrated a linear relationship between fat mass and arterial compliance that was continuous in girls across the range of measured values but discontinuous in boys, becoming positive only in boys with more than 24 kg of fat. The current study demonstrates that differences in body composition, especially fat mass in obese children, exert a strong influence on vascular compliance in children. Further studies are needed to determine the underlying mechanisms of action and the long-term implication of obesity on vascular health and disease risk in children.

## Figures and Tables

**Figure 1:**
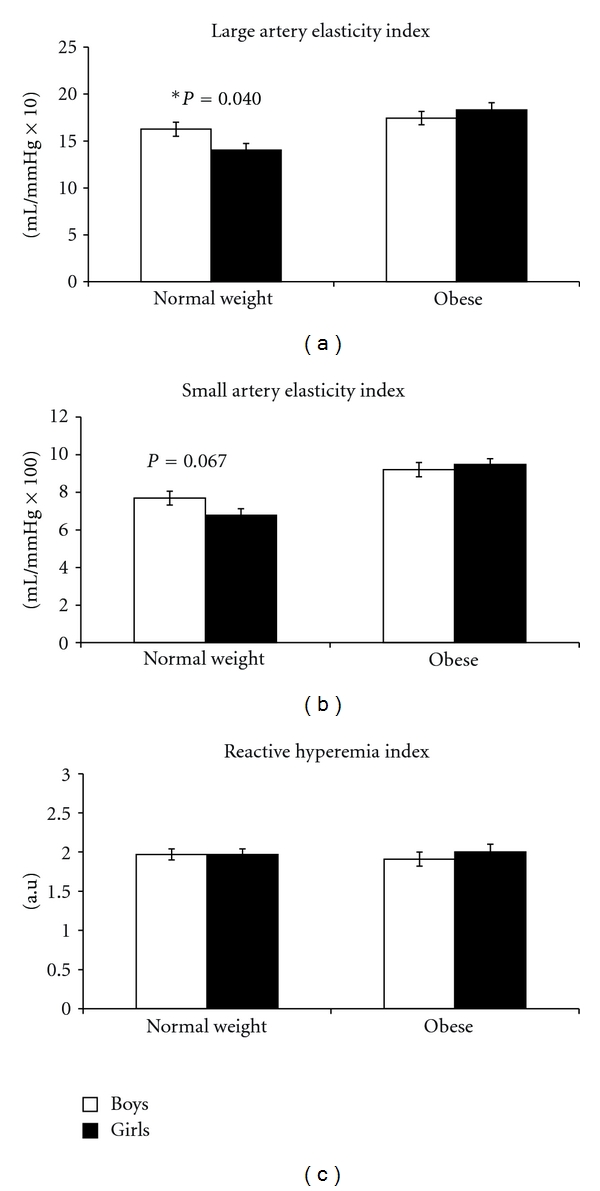
Reactive hyperemia index (RHI) and large and small arterial elasticity index (LAEI and SAEI, resp.) in boys and girls. Values are for mean ± SEM of normal-weight and obese groups. LAEI was significantly increased in the normal-weight boys compared to the normal-weight girls. SAEI was not different in boys and girls, but in the normal-weight group, the difference approached significance (*P* = 0.067). There was not a sex difference for RHI in either weight group. *Boys > girls in the normal-weight group (*P* < 0.05).

**Table 1:** Subject characteristics.

	Normal-weight boys (*N* = 30)	Normal-weight girls (*N* = 31)	Obese boys (*N* = 30)	Obese girls (*N* = 32)
Age (years)	13.3 ± 2.6	13.4 ± 3.4	13.7 ± 2.4	14.1 ± 2.6
Height (cm)	158.6 ± 17.0	150.4 ± 16.6	163.7 ± 13.8	160.5 ± 11.3
Weight (kg)	48.3 ± 13.4	45.3 ± 13.8	84.7 ± 25.1	86.6 ± 20.1
Lean mass (kg)	34.2 ± 11.1*	27.0 ± 8.2	44.2 ± 13.4	42.5 ± 9.3
Fat mass (kg)	9.7 ± 3.1	13.4 ± 5.1*	34.0 ± 14.3	39.7 ± 12.7
Tanner stage	2.8 ± 1.4	2.9 ± 1.2	3.0 ± 1.0	3.7 ± 1.0*
Systolic blood Pressure (mmHg)	109 ± 8	107 ± 8	118 ± 8	117 ± 8
Diastolic blood Pressure (mmHg)	57 ± 6	58 ± 5	59 ± 6	61 ± 7

All measures are mean ± SD. *Denotes difference between boys and girls within groups defined by obesity where *P* < 0.05.

**Table 2:** Serum Measures.

	Normal-weight boys (*N* = 30)	Normal-weight girls (*N* = 31)	Obese boys (*N* = 30)	Obese girls (*N* = 32)
Glucose (mmol/L)	4.66 ± 0.33	4.61 ± 0.39	4.77 ± 0.33	4.61 ± 0.44
Insulin (pmol/L)	43.06 ± 29.86	51.39 ± 48.62	127.79 ± 84.73	192.38 ± 153.48*
HOMA-IR	1.29 ± 0.87	1.57 ± 1.54	3.91 ± 2.72	5.92 ± 5.84
Cholesterol (mmol/L)	40.15 ± 6.22	42.22 ± 7.51	53.87 ± 24.35	46.88 ± 12.17
HDL-C (mmol/L)	12.30 ± 2.67	12.79 ± 2.85	12.20 ± 3.57	12.35 ± 3.39
LDL-C (mmol/L)	23.18 ± 5.80	24.42 ± 6.92	35.90 ± 22.04	30.02 ± 10.41
Triglycerides (mmol/L)	0.74 ± 0.27	0.89 ± 0.41	1.47 ± 0.79*	1.10 ± 0.50
CRP (mg/L)	0.72 ± 1.37	0.76 ± 1.30	2.17 ± 2.56	4.13 ± 6.06

All measures are mean ± SD. *Denotes difference between boys and girls within groups defined by obesity where *P* < 0.05.

**Table 3:** Univariable correlations with LAEI.

Group	Correlate	*r*-value	*P* value
Normal-weight boys	Height	0.73	<0.0001
	Lean mass	0.69	0.0001
	Age	0.61	0.0003
	Tanner stage	0.54	0.04

Normal-weight girls	Height	0.63	0.0001
	Lean mass	0.60	0.0004
	Fat mass	0.57	0.0008
	Tanner stage	0.56	0.01
	Age	0.42	0.002

Obese boys	Height	0.70	<0.0001
	Lean mass	0.66	0.0002
	Age	0.66	0.0003
	Tanner stage	0.56	0.02
	Fat mass	0.54	0.02

Obese girls	Fat mass	0.51	0.003
	Height	0.49	0.01
	Lean mass	0.48	0.02

**Table 4:** Univariable correlations with SAEI.

Group	Correlate	*r*-value	*P* value
Obese boys	Fat mass	0.68	0.0001
	Lean mass	0.65	0.0005
	Height	0.61	0.002
	Age	0.52	0.001

Obese girls	Tanner stage	0.55	0.02
	Fat mass	0.46	0.007
	Lean mass	0.40	0.02
